# 269. Reference Data of Biochemical and Hematology Parameters in Juvenile and Adult Owl Monkeys (*Aotus nancymaae*) for Biomedical Research

**DOI:** 10.1093/ofid/ofad500.341

**Published:** 2023-11-27

**Authors:** Juan Carlos Gaspar, Tyler Moeller, Jessica Buchta, Monica Nieto, Nereyda Espinoza, Rosa Castillo, Rosa Nunez

**Affiliations:** Naval Medical Research Unit 6, Lima, Lima, Peru; Naval Medical Research Unit 6, Maryland, Maryland; Naval Medical Research Unit 6, Lima, Lima, Peru; Naval Medical Research Unit 6, Lima, Lima, Peru; Naval Medical Research Unit 6, Lima, Lima, Peru; Naval Medical Research Unit 6, Lima, Lima, Peru; Naval Medical Research Unit 6, Lima, Lima, Peru

## Abstract

**Background:**

Non-human primates (NHP), including New World monkeys (NWM), are used in varied fields of biomedical research encompassing infectious disease, behavioral sciences and drug and vaccine development. In these studies, normal reference values for biochemical and hematological parameters are often needed for comparison with experimental results. These blood parameters are useful guides in reaching a diagnosis, estimating the prognosis of the disease and monitoring the progress of treatment. Although there are various reports of biochemical and hematologic parameters in Old World monkeys, the range of reference values often differs between NHP species. Despite the importance of NWMs such as *Aotus nancymaae* in biomedical research, few studies have examined these values and with a limited number of animals only. *A. nancymaae* is widely used for research and often used to study the principal types of human malaria (*P. falciparum* and *P. vivax*). These primates have also been used to study leishmaniasis, schistosomiasis, tuberculosis and various types of enteric infections. Furthermore, in recent years oral challenge models have been developed in *A. nancymaae* for *Campylobacter jejuni*, *Shigella flexneri*, and Enterotoxigenic *Escherichia coli* (ETEC). However, there is no updated data on biochemical and hematological parameters for *A. nancymaae*. Therefore, the aim of this study was to provide complete and accurate reference intervals of biochemical and hematological values to assess the health status indices of *A. nancymaae* housed in a vivarium prior to their use.

**Methods:**

To characterize these parameters, a total of 1250 blood samples (1003 from juvenile and 247 from adult monkeys) were analyzed between 2007 to 2022. A total of 14 biochemical and 20 hematology parameters were measured.

**Results:**

A significant change was observed in the values of alkaline phosphatase (ALP), potassium (K^+^), glucose (GLU), blood urea nitrogen (BUN), creatinine (CRE), hemoglobin (HGB) and lymphocyte (LYM) in relation to sex and age.

**Conclusion:**

In conclusion, this work establishes a comprehensive and systematic reference of serum biochemical and hematological parameters for *A. nancymaae* based on a large cohort which can be used to inform and guide future studies utilizing this animal model.

**Disclosures:**

**All Authors**: No reported disclosures

